# Physical and digital safety for older adults with Parkinson's disease: Reflections from an interdisciplinary community programme

**DOI:** 10.51866/mol.26-00050

**Published:** 2026-10-03

**Authors:** Hasliza Abu Hassan, Muhammad Fairuz Abd Rauf, Mohd Fahmi Mohamad Amran

**Affiliations:** 1 Primary Care Medicine Department, Faculty of Medicine and Defence Health, Universiti Pertahanan Nasional Malaysia, Kem Perdana Sungai Besi, Malaysia.; 2 Computer Science Department, Faculty of Science and Defence Technology, Universiti Pertahanan Nasional Malaysia, Kem Perdana Sungai Besi, Malaysia.

**Keywords:** Health education, Parkinson disease, Falls, Internet

Patient safety in older adults is often viewed clinically through medication review, home hazards, mobility and caregiver support. A community programme between Universiti Pertahanan Nasional Malaysia and the Malaysian Parkinson’s Disease Association (MPDA) widened this perspective by addressing digital safety and independence among older adults. Conducted on 25 February 2025, the programme provided education on fall prevention and internet safety for people with Parkinson’s disease (PD) and their caregivers.

**Lessons from the community**

The fall prevention session became more meaningful when participants related the advice to their daily challenges. Several used walking aids or wheelchairs, while others described balance problems and fear of falling. Suggestions such as improving lighting, removing loose rugs, using appropriate footwear and remaining active were useful when adapted to each person’s home, mobility and support. Their questions reminded us that practical advice must fit individual circumstances.

The internet safety session highlighted a different form of vulnerability. Online banking, messaging applications and digital services help older adults remain connected, but they may also expose them to scams, phishing and impersonation. Participants wanted more than advice on recognising suspicious messages. They asked what they should do after opening a suspicious link, sharing personal details or transferring money. These questions made us realise that digital harm is not only about financial loss. It may also lead to anxiety, embarrassment, loss of confidence and a sense of lost dignity.

Participants also expressed the need for better advocacy for older adults and more collaboration with universities. Their comments reminded us that a single educational session has its limitations. Follow-up activities may help reinforce what participants have learnt and address new concerns.

**Reflective insights**

The sessions became especially meaningful through the two-way interactions with participants. While we shared professional knowledge, participants shared their experiences of living with PD. This experience reminded me, as a family physician, that safety in older adults should not be viewed only from a clinical perspective. I usually associated patient safety with falls, medication use, mobility and the home environment. The internet safety discussion made me more aware that digital risks may also affect an older person’s independence, emotional well-being and quality of life.

This experience also reminded me that advice given in the clinic may not always fit into a patient’s daily life. Understanding a patient’s needs and risks requires consideration of their clinical condition, functional ability, home environment, caregiver support and use of digital services. Working with colleagues from a different field strengthened my understanding on how health and digital safety are closely related in older adults’ daily lives. Direct engagement with the community may also help us better understand their concerns and needs.

The programme reinforced that caring for older adults goes beyond preventing physical harm. The identified emerging risks are best addressed through shared awareness among healthcare professionals, caregivers, community members and government agencies. Digital safety may therefore be considered within holistic primary care, as part of a wider shared effort to support older adults’ financial security, emotional well-being and overall quality of life.

**Figure:**
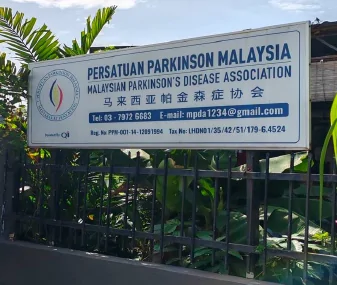
a) Venue of the programme.

**Figure:**
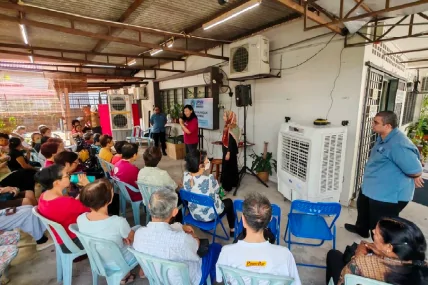
b) President of the MPDA delivering the opening remarks.

**Figure:**
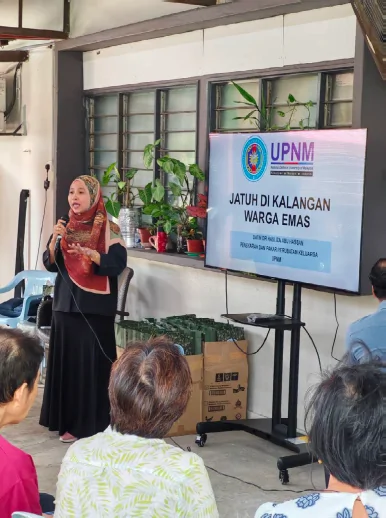
c) Datin Dr Hasliza delivering the session on fall prevention.

